# A Case of COVID-19 Re-Infection in a Liver Transplant Patient

**DOI:** 10.7759/cureus.14916

**Published:** 2021-05-09

**Authors:** Michael Mohseni, Michael Albus, Ann Kaminski, Michael F Harrison

**Affiliations:** 1 Emergency Medicine, Mayo Clinic, Jacksonville, USA

**Keywords:** coronavirus, sars-cov-2, covid-19, transplant, re-infection, immunosuppression

## Abstract

Coronavirus disease 2019 (COVID-19) is an ongoing worldwide pandemic infection. The exact incidence of disease re-infection or recurrence remains unknown. One particular at-risk population includes individuals with solid organ transplantation on immunosuppression. We present a case of COVID-19 re-infection in a chronically immunocompromised liver transplant patient.

A 53-year-old female presented to the Emergency Department (ED) with nausea, vomiting, diarrhea, and myalgias. She was found to test positive for COVID-19. Her relevant medical history included liver transplantation on chronic immunosuppression. More recently, she had tested positive for COVID-19 approximately three months prior to this and was hospitalized at that time for encephalopathy and treated with remdesivir and convalescent plasma. She had subsequently recovered with negative COVID-19 testing in the interim. On the ED presentation with presumed re-infection, her disease was deemed to be mild with lack of severe symptoms or pulmonary involvement, and she was discharged with outpatient follow-up for monoclonal antibody infusion therapy.

We describe a scenario of presumed COVID-19 re-infection in a liver transplant patient. To our knowledge, this is a rare event and has been reported internationally in only a handful of individuals. We surmise that immunosuppression could offer some protection from the inflammatory cascade of the initial disease process in COVID-19 given the relatively mild disease observed in our patient. On the other hand, a less robust immune response may decrease humoral immunity and leave patients at greater risk of re-infection. Further investigation is necessary to delineate COVID-19 disease re-infection versus relapse, especially in the setting of an immunocompromised state.

## Introduction

The first case of severe acute respiratory syndrome coronavirus (SARS-CoV-2), commonly referred to as coronavirus disease 2019 (COVID-190, was diagnosed in China in late 2019 [1]. Since that time, COVID-19 has become a global pandemic with more than 42 million confirmed cases [2-3], and, in the United States, it is responsible for more than 400,000 deaths [4]. At-risk populations have been identified and commonly include adults older than 65 years of age or adults of any age with a history of cancer, chronic kidney disease, chronic lung disease such as chronic obstructive pulmonary disease (COPD), obesity, hypertension, diabetes mellitus, and immunocompromising conditions such as HIV and solid organ transplantation [5-6].

There is controversy with respect to the degree and duration of immunity afforded to an individual by a previous COVID-19 infection. Given the novelty of COVID-19 infection, the exact degree and duration of protection is not yet fully understood. However, it has been shown that previous infection offers some protection for at least four to five months in immunocompetent hosts [7-9]. Transplant recipients in general and liver transplant recipients specifically have been reported to experience lower prevalence and less disease severity with respect to COVID-19 [10-12]. The decreased prevalence has been attributed to conscious behaviors to avoid exposure and reduce the risk of contracting COVID-19 among individuals of this at-risk population. However, this paradoxical finding of decreased severity may be attributed to an “ideal” level of immunosuppression in post-transplant patients that favorably modulates the immune and inflammatory reactions to COVID-19 infection. Though data related to immune response are sparse, one case series suggests that seroconversion does not readily occur in liver transplant patients [13]. COVID-19 reinfection represents a rare event, but episodes of recurrent infection have been reported in the kidney transplant population [14-15]. However, cases of reinfection among liver transplant recipients are not readily identifiable in the current published literature. We present a case of COVID-19 reinfection in a chronically immunocompromised liver transplant patient.

## Case presentation

A 53-year-old female presented to our Emergency Department (ED) in January 2021 with a six-day history of nausea, vomiting, diarrhea, and myalgias. Her relevant past medical history included liver transplant in 2010 due to alcoholic cirrhosis, hypertension, hypothyroidism, anxiety, and chronic kidney disease. She also reported that she had been diagnosed with and treated for COVID-19 infection three months prior to the current ED visit in October 2020. She was hospitalized at that time for encephalopathy due to her COVID-19 infection and was treated with remdesivir and convalescent plasma. No virus serotyping was done on this admission. She recovered and was discharged from the hospital after a seven-day course that did not involve admission to the intensive care unit or require any significant supplemental oxygen therapy beyond standard nasal cannula. She received a negative COVID-19 result approximately one month after discharge from the hospital in November 2020 and reported a full recovery in the interim. Her immunosuppression regimen was tacrolimus 1 mg twice daily, and her graft function since transplantation was stable with normal results on outpatient laboratory and radiographic monitoring.

During the present ED encounter (January 2021), she was stable from a hemodynamic and respiratory perspective with normal vital signs. Physical examination revealed no acute abnormalities, including normal lung examination. She received a positive COVID-19 result (SARS-CoV-2 Rapid PCR kit, Roche Diagnostics, Rotkreuz, Switzerland). The remainder of her laboratory workup revealed no significant derangements other than mild baseline renal insufficiency with a creatinine of 1.28 mg/dL.

Her nausea and vomiting were not intractable, and after consultation with the hospitalist service, no reason was identified for inpatient management, especially in light of lack of severe symptoms or pulmonary involvement. She was discharged from the ED and referred to our outpatient service for monoclonal antibody infusion therapy and was also instructed to follow up with her primary care and transplant providers.

## Discussion

We presented a case of COVID-19 re-infection in a liver transplant patient. To the best of our knowledge, this is the first report of recurrent infection in a liver transplant recipient in the United States (as of the writing of this report). Fortunately, for our patient, the course of both infections was mild. The initial infection required hospitalization due to altered mentation, but she was managed with minimal supplemental oxygen on the general hospital floor. The patient’s re-infection was evaluated in the ED and did not require hospitalization. Surprisingly, this mild clinical course may not be that unusual for liver transplant patients based on the available literature [10-12].

In general, immunosuppression increases the risk of viral infections [16]. Research into the pathophysiology of the COVID-19 disease process is ongoing, but it appears that patients are at risk from the effects of the virus as well as from the effects of the host’s inflammatory cascade [17-18]. In solid organ transplant patients receiving immunosuppression, there is some evidence that immunosuppression attenuates the body’s inflammatory response as evidenced by decreased interleukin (IL)-6 blood values [19]. This immunosuppression could offer some protection from the inflammatory cascade of the initial disease process. Our patient’s monotherapy with tacrolimus for immunosuppression may have been associated with the observed decreased severity of her COVID-19 illnesses. On the other hand, a less robust immune response may decrease humoral immunity and leave patients at a greater risk of re-infection. The American Association for the Study of Liver Diseases (AASLD) has provided consensus statements on both outpatient and inpatient management of COVID-19 patients with liver transplantation. In outpatients, the AASLD does recommend the use of monoclonal antibodies that target the SARS-CoV-2 spike protein to help decrease the need for possible hospitalization and potentially death by decreasing the viral load [20]. This is in line with the therapy recommended for our patient during her recurrent COVID-19 illness given that she was deemed stable for outpatient management. With regard to the severity of illness in COVID-19 liver transplant patients, older individuals (60 years or older) and those with diabetes mellitus or hypertension are at increased risk of mortality [21]. In some larger case series, mortality rates as high as 19% have been observed in liver transplant patients with COVID-19 [22]. Fortunately, for our patient, her younger age and lack of diabetes mellitus likely protected her from more severe disease manifestations.

Overall, COVID-19 re-infection appears to be a rare event. In the setting of liver transplantation, to our knowledge, this has only been reported internationally (Egypt) in two other individuals [23]. Also, there remains debate on the possibility of prolonged nucleic acid conversion, suggesting that some patients may test falsely negative during recovery and subsequently appear to “turn positive” once again [24]. In our case report, given the patient’s three-month symptom-free interval, we surmise that re-infection occurred. What remains unknown is whether this clinical picture might also be explained by persistence of nonviable RNA after the first COVID‐19 episode or by COVID‐19 relapse, especially in an immunosuppressed patient [25]. Future investigations are warranted to better delineate the clinical picture and course of patients who present with apparent repeat COVID-19 infections.

## Conclusions

We presented a case report highlighting presumed COVID-19 re-infection in a liver transplant patient. To our knowledge, this a rare event and has been previously reported in only a handful of patients with liver transplantation. Further investigation is necessary to delineate COVID-19 disease re-infection versus relapse, especially in the setting of an immunocompromised state.
